# Supplementary material for: Patterns of Intron Gain and Loss in Fungi
Source: PLoS Biol. 2004 Nov 30;2(12):e422. doi: 10.1371/journal.pbio.0020422 (PMC532390; doi:10.1371/journal.pbio.0020422)
Supplement: Table S1 — Also available at http://genes.mit.edu/NielsenEtAl/. (4.3 MB ZIP). [file pbio.0020422.st001.zip › NielsenEtAl/html/130.html]

AN5751.1.NCU08151.1.MG09734.1.FG10482.1


```
 CLUSTAL W (1.82) Multiple Sequence Alignments - Introns Inserted


Sequence 1: MG09734.1	538 aa
Sequence 2: FG10482.1	458 aa
Sequence 3: NCU08151.1	487 aa
Sequence 4: AN5751.1	519 aa
Alignment Length: 645 aa
Number Identitical Residues: 133 aa
Alignment Score (without introns) 7999


MG09734.1 	MSSPG--KRRASNAAISPPPIKRKAQTTIT~K1TAVASFFTP---TSQ~KPKERTTWNER
NCU08151.1	MGPTKR-PAEEGDRSISPPPLKRKAQTAIS~S~----SLHCPSFFCYA0KPKDRTTWTEK
FG10482.1 	MSSDDQPKRKASDAPVSPPPIKRKVQSGTT~T1----NFFTP---TSQ~KPKDPTVWSER
AN5751.1  	MAGEGALKRAAS-RSISPPPLRRKAETLKK1S~-VATSFFTP---VSQ~RKTEPTSISWR
          	*.         .  .:****::**.::  . .    :.:. *       : .: *  . :

MG09734.1 	MPPKDGKDSNDARATLLVAQYTPE-----SYEAEPPSKRRKVAAFDL~DGTIIRTASGKK
NCU08151.1	SP------DADSPATLLVAKYVPEGSPTNDESVNTTVKRRKIAAFDL0DSTLITSASGKK
FG10482.1 	SP----NNNDDIPATLLVGRFEPE-----KKEDRK-VKRRKIAAFDL~DSTLISTSSGKK
AN5751.1  	IVN----------HTLIVGKFSLG----PGEPPPKSSGKPKIAAFDF0DSTLVATASGNT
          	              **:*.::              .  : *:****: *.*:: ::**:.

MG09734.1 	HADGPGDWQWWDTCVPLKLKSLYYEDG2YRVVIFSNQGGLTLHPDP-------KSKGPKN
NCU08151.1	HSHDAADWKWWHHSVPDRLRKLYNEEG2YQVIIFTNQGGLTLHASPSSSSSSSKPKTPKA
FG10482.1 	HASSGTDWKWWHSSVPTKLRELY-QDG~YRVVILSNQAGLTLHFDA-------KHKGPKA
AN5751.1  	FPRDSADWKWWRQNVPSRLQKLN-ADG2YHVVIFTNQGKISLKKDK------------KG
          	.. .  **:**   ** :*:.*   :* *:*:*::**. ::*: .             * 

MG09734.1 	--TKRTDQFKTKVNSILSKLDIPITLYAATAKDIFRKPRPGMWNEMLNDYDLNGTEATPE
NCU08151.1	Q-LDRVPQFKQKCSAVLSQLDIPTTLYAATGKDIYRKPRPGMWLEMKADYNLFN-DDDID
FG10482.1 	NAQKRVSEFKQKCSAVLNSLNLPTCVYAATEHDIYRKPRIGMWKEVCEDYDIPETEVD--
AN5751.1  	NVSSYFNKFKERVSAVMKQLNIPLSVYAATEHDEYRKPRAGMWKEFLDDYDFDVTGID--
          	.  .   :** : .:::..*::*  :**** :* :**** *** *.  **::  :     

MG09734.1 	MEHSFFVGDAGGRTAQLVSASAVTANAAIKSKGKGRAGPAALPKDFSCSDR~NLAHNIGI
NCU08151.1	LENSIFVGDAGGRQSELPP------------NSNGRIKATATPKDFSCSDR~NLAHNVGI
FG10482.1 	LEKSIFVGDAGGRTAG---------------VGKGPDGVAAMSKDFSCSDR~NFAHNAGI
AN5751.1  	SSQSIFVGDAAGR-----------------------------PGDHSAADR2GFAANANM
          	 .:*:*****.**                             . *.*.:** .:* * .:

MG09734.1 	DFKTPEEYFLGEEPRDFVRDFDLASHPFP------DAD--GEP~KALIE------QKNEK
NCU08151.1	QYQTPEEFFLGEEPRNFTRDFDLVKYPYPSSPTTTDPDSSSSS~SSSKKEEILFTKTSPQ
FG10482.1 	KFMTPEEFFLGEKARSYAREFDLAEHPFS------DDTSSGSS~VVTLK------RTNDQ
AN5751.1  	VFKTPEEFFLGAAP-EPVVAFDPAVYLQN--------NSVDDA1LPQFS------KTSPL
          	 : ****:***  . . .  ** . :            : ...     .      :..  

MG09734.1 	DIILFCGPPGAGKSTFYWKYLKPLGYGRVNQDTLKT2KEKCLSAAAEMLKDKVSVVID1N
NCU08151.1	ELVLFVGPPGAGKSTFYWRHLKPLGFERVNQDVLKS~KDKCLKAATEYLKEGDSVVVD~N
FG10482.1 	DIILFCGPPGAGKSTLYWKSLKPLGYARINQDLLKT~RDKCVQAAKEHLQEGTSVAID1N
AN5751.1  	ELVIFCGSPGAGKSTFFWDYLKPLGYERVNQDILKS0RAKCIKVAKEHLAAQKSVVVD1N
          	::::* *.*******::*  *****: *:*** **: : **:..* * *    **.:* *

MG09734.1 	TNPDPDTRALWVALAKKHDIPIRCVWFKTPLALAQHNDAVRSMNGT0L~QLETQGAKG1T
NCU08151.1	TNPDPDTRKQWVELAKKQGVPVRCVWFRTPLVVCEHNDAVRALNKP0L~NPESR----~T
FG10482.1 	TNADPATRTVWVELAKEFGISIRCVWFKTPLQVCEHNNAVRALNES~L~NPESR----~L
AN5751.1  	TNADVETRAQWIGVAKEFNVPIRCVYFTAPPALCKHNNAVRAANTS~L0NPESR----~T
          	**.*  **  *: :**: .:.:***:* :*  :.:**:***: * . * : *::      

MG09734.1 	ITPDHHLDRYAAAVEWKRAWYAETEATRTATAATVVTASEAHFGFISTA0VSESLLLKAK
NCU08151.1	SLPKLAFNSFNS-------------------------------RFKEPK~VKEGFQDVTE
FG10482.1 	VLPKMAFTSFAN-------------------------------RFKPPS~VKEGFQDIIE
AN5751.1  	LLPGIAFGDFVK-------------------------------RFKEPS~LAEGFQDILR
          	  *   :  :                                  *  .  : *.:    .

MG09734.1 	IPGRASIPIVSSRTGCSLTSTAAAGVCSGI~LFLSSMFDDILRLA-----------~---
NCU08151.1	VD-------------FKFRGTKEEYEIWGK~YWI----------------------~---
FG10482.1 	VP-------------FQFRGTEEEYRIWGR~YWT----------------------~---
AN5751.1  	VD-------------FRFRGDDDAKNLWKH2QMILCTTLLSPGSAPPSASIVRPRY0VEK
          	:                : .               .        :..:::   .    ..

MG09734.1 	------------------------------------------------~-----------
NCU08151.1	------------------------------------------------~-----------
FG10482.1 	------------------------------------------------~-----------
AN5751.1  	LSSYGLLLALLCSAATEHATSADLPIGRGVTSCQTLDFVPTRRDLTAA1SKEVQASQGPI
          	 :: .   :  .::::. ::::. . . . ::..: .  .:  . ::: :.. .::... 

MG09734.1 	-
NCU08151.1	-
FG10482.1 	-
AN5751.1  	I
          	
```
